# Airway antibodies emerge according to COVID-19 severity and wane rapidly but reappear after SARS-CoV-2 vaccination

**DOI:** 10.1172/jci.insight.151463

**Published:** 2021-11-22

**Authors:** Alberto Cagigi, Meng Yu, Björn Österberg, Julia Svensson, Sara Falck-Jones, Sindhu Vangeti, Eric Åhlberg, Lida Azizmohammadi, Anna Warnqvist, Ryan Falck-Jones, Pia C. Gubisch, Mert Ödemis, Farangies Ghafoor, Mona Eisele, Klara Lenart, Max Bell, Niclas Johansson, Jan Albert, Jörgen Sälde, Deleah D. Pettie, Michael P. Murphy, Lauren Carter, Neil P. King, Sebastian Ols, Johan Normark, Clas Ahlm, Mattias N. Forsell, Anna Färnert, Karin Loré, Anna Smed-Sörensen

**Affiliations:** 1 Division of Immunology and Allergy, Department of Medicine Solna, Karolinska Institutet, Karolinska University Hospital, Stockholm, Sweden.; 2 Unit of Biostatistics, Institute of Environmental Medicine, and; 3 Department of Physiology and Pharmacology, Karolinska Institutet, Stockholm, Sweden.; 4 Department of Perioperative Medicine and Intensive Care, Karolinska University Hospital, Stockholm, Sweden.; 5 Division of Infectious Diseases, Department of Medicine Solna, Center for Molecular Medicine, Karolinska Institutet, Stockholm, Sweden.; 6 Department of Infectious Diseases, Karolinska University Hospital Solna, Stockholm, Sweden.; 7 Department of Microbiology, Tumor and Cell Biology, Karolinska Institutet, Stockholm, Sweden.; 8 Division of Clinical Microbiology, Karolinska University Laboratory, and; 9 Närakut SLSO, Karolinska University Hospital Solna, Stockholm, Sweden.; 10 Department of Biochemistry and; 11 Institute for Protein Design, University of Washington, Seattle, Washington, USA.; 12 Section of Infection and Immunology, Department of Clinical Microbiology, Umeå University, Umeå, Sweden.

**Keywords:** COVID-19, Immunology, Adaptive immunity, Immunoglobulins, Innate immunity

## Abstract

Understanding the presence and durability of antibodies against SARS-CoV-2 in the airways is required to provide insights into the ability of individuals to neutralize the virus locally and prevent viral spread. Here, we longitudinally assessed both systemic and airway immune responses upon SARS-CoV-2 infection in a clinically well-characterized cohort of 147 infected individuals representing the full spectrum of COVID-19 severity, from asymptomatic infection to fatal disease. In addition, we evaluated how SARS-CoV-2 vaccination influenced the antibody responses in a subset of these individuals during convalescence as compared with naive individuals. Not only systemic but also airway antibody responses correlated with the degree of COVID-19 disease severity. However, although systemic IgG levels were durable for up to 8 months, airway IgG and IgA declined significantly within 3 months. After vaccination, there was an increase in both systemic and airway antibodies, in particular IgG, often exceeding the levels found during acute disease. In contrast, naive individuals showed low airway antibodies after vaccination. In the former COVID-19 patients, airway antibody levels were significantly elevated after the boost vaccination, highlighting the importance of prime and boost vaccinations for previously infected individuals to obtain optimal mucosal protection.

## Introduction

SARS-CoV-2 infection that causes COVID-19 presents with a wide range of disease severity, from asymptomatic to fatal (1, 2). Individuals of advanced age and/or those with comorbidities are overrepresented among patients who develop severe disease (3). However, the majority of SARS-CoV-2–infected individuals experience asymptomatic infection or only mild disease (4).

Systemic antibodies against the SARS-CoV-2 nucleocapsid (N) and the viral surface glycoprotein spike (S) as well as against the receptor binding domain (RBD) (5, 6) of the S protein have been studied extensively (7–11). Responses against the internal N protein are often readily detectable, but their contribution to protection and control of disease is not clear (8, 10). In contrast, antibody responses against S and, in particular, against the RBD result in virus neutralization (12). Responses against the RBD are thus likely necessary for protection from reinfection or prevention of symptomatic disease. However, the presence and durability of antibodies during COVID-19 in the airways is still not well understood.

The respiratory tract is the initial site of viral infection and replication. The availability of antibodies at this site could therefore determine the ability to neutralize the virus locally in case of (re-) exposure and prevent viral spread. Generally, antibodies present in the circulation and at local sites are the result of secretion from short-lived plasmablasts and/or terminally differentiated plasma cells in the bone marrow or mucosal sites (13). However, the response to a secondary infection once antibody titers have waned below protective levels mostly relies on the presence of resting antigen-specific memory B cells that are rapidly activated upon antigen reexposure (13). Whether vaccination against SARS-CoV-2 also elicits systemic antibody responses in addition to local antibodies in the airways of individuals who recovered from COVID-19, and via which mechanism, are currently unknown.

In this study we present data from a cohort of patients that we have followed since mid-March 2020, which was the start of the pandemic in Sweden. We show longitudinal data on virus-specific systemic and airway antibody and B cell memory responses generated in this clinically well-characterized cohort of individuals with SARS-CoV-2 infection (*n* = 147) ranging from asymptomatic SARS-CoV-2 infection to fatal COVID-19. In addition, we show how subsequent SARS-CoV-2 vaccination during the convalescent phase significantly boosts not only systemic but also airway antibody responses.

## Results

### Patient enrollment, assessment of disease severity, and timeline.

**Figure 1:**
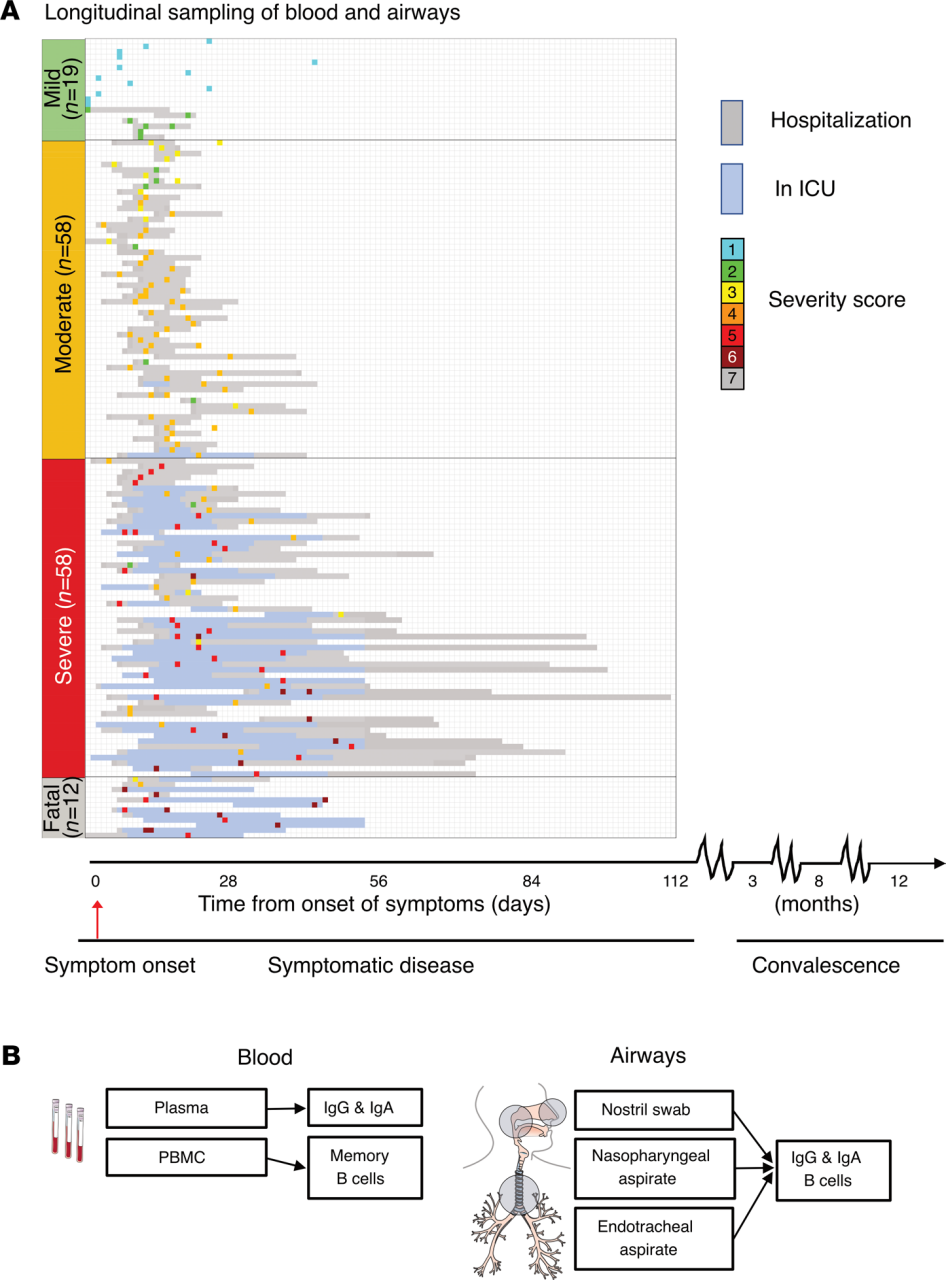
Study and sampling overview. (**A**) Overview of study cohort (*n* = 147), timeline of longitudinal sampling, hospital admission/discharge, level of care, and outcome for each patient. Patients are grouped based on peak disease severity (PDS): mild (PDS 1 and 2), moderate (PDS 3 and 4), severe (PDS 5 and 6), and fatal (PDS 7). Individual inclusion sample for each patient is color-coded based on disease severity at the time of sampling. (**B**) Overview of the anatomical compartments analyzed and the measurements performed.

Individuals were sampled longitudinally in blood and airways during acute infection/symptomatic disease and during convalescence (median 3 and 8 months from symptom onset). Donor-matched plasma, peripheral blood mononuclear cells (PBMCs), nostril swabs (NSWs), and nasopharyngeal aspirates (NPAs) were collected from all patients across disease severities whereas endotracheal aspirates (ETAs) were only collected from intubated patients receiving intensive care (Figure 1). Disease severity was assessed daily, using a 7-point scale derived from the respiratory domain of the sequential organ failure assessment (SOFA) score (14, 15), with additional levels for nonadmitted and fatal cases (Table 1). Patients were grouped based on peak disease severity, which may differ from disease severity at the time of sampling (Table 1 and Figure 1B). In addition, prepandemic healthy controls (PPHCs) (*n* = 30) as well as individuals with influenza-like symptoms, and possible SARS-CoV-2 exposure, but negative diagnostic PCR results (PCR^–^) (*n* = 9) were sampled in the same way and included as controls. Generally, severe patients were sampled later after symptom onset as compared with individuals with mild disease, resulting in a large time frame of study inclusion with respect to symptom onset (Table 1 and Figure 1B) (16). For simplicity, the sampling period/study inclusion during ongoing infection and hospitalization (for those hospitalized) is referred to as the “acute” phase. Samples collected at the first follow-up visit during convalescence (range 46–168 days from symptom onset; median 108 days, coefficient of variation 21.56%) are referred to as the 3-month time point whereas those collected at the second follow-up visit (range 187–344 days; median 245 days, coefficient of variation 9.52%) are referred to as the 8-month time point. Time of the first convalescent follow-up sampling from acute sampling ranged 33–159 days; median 90 days, coefficient of variation 24.84% (Table 1).

### Plasma IgG and IgA responses to N, S, and RBD across COVID-19 severity during acute disease and after recovery.

**Figure 2:**
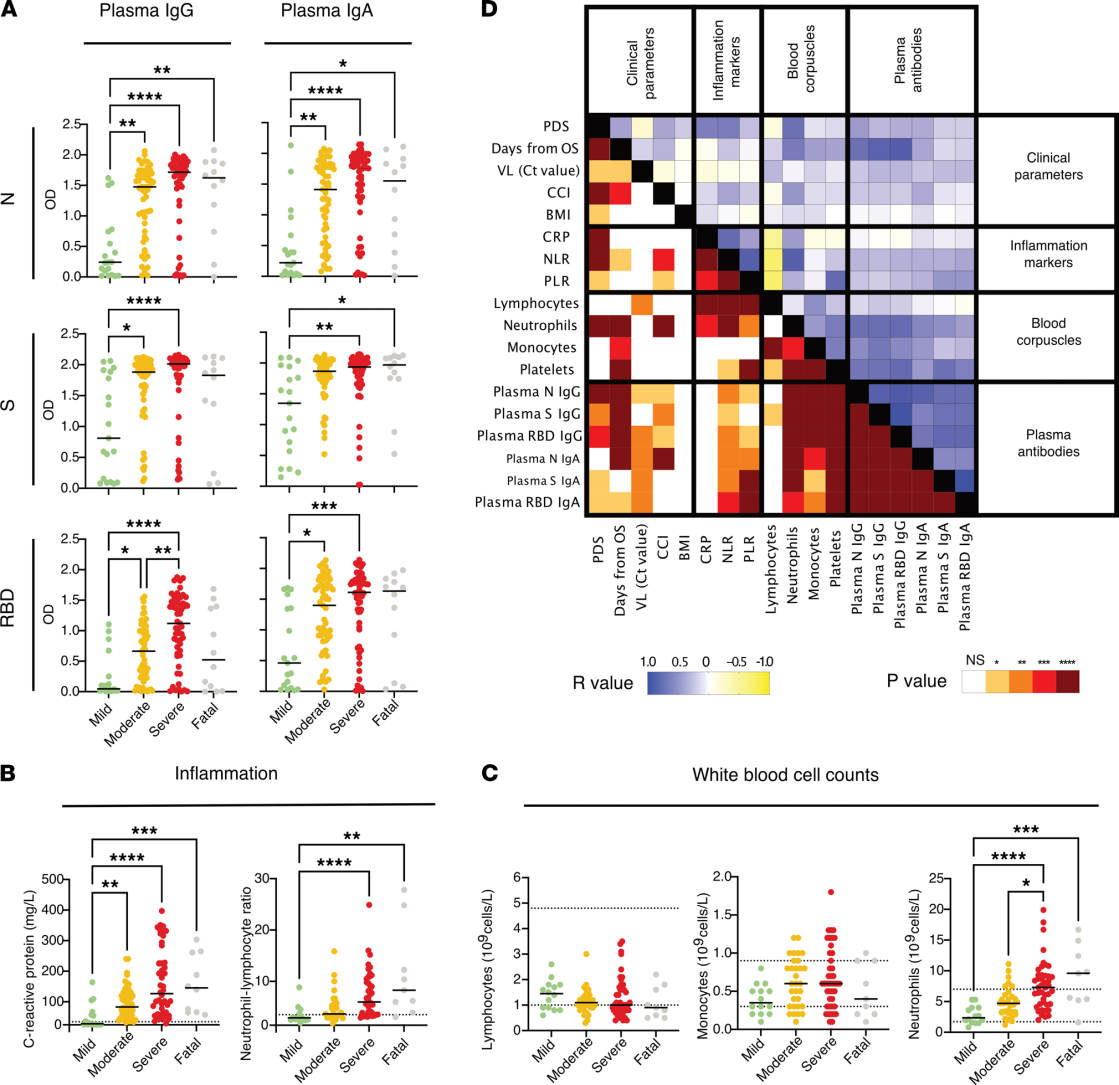
Systemic antibody responses, inflammation markers, and other clinical parameters in relation to COVID-19 severity during acute disease. (**A**) Plasma IgG and IgA responses (*n* = 19 for mild, *n* = 58 for moderate, *n* = 58 for severe, and *n* = 12 for fatal) against N, S, and RBD are shown together with the levels of (**B**) CRP and the NLR as a measure of systemic inflammation and with (**C**) the levels of lymphocytes, monocytes, and neutrophils. Black lines indicate medians. Differences were assessed using Kruskal-Wallis with Dunn’s multiple comparisons test and considered statistically significant at *P* < 0.05. *P < 0.05, **P < 0.01, ***P < 0.001, ****P < 0.0001. The dashed lines indicate the normal thresholds or range values. (**D**) Correlation matrix summarizing the interrelationship observed between the clinical parameters, inflammation markers, blood corpuscles, and data from systemic antibody levels measured during acute disease as indicated. The *P* and *R* values (Spearman) are shown separately in the mirrored halves of the matrix and have been color-coded as indicated.

**Figure 3:**
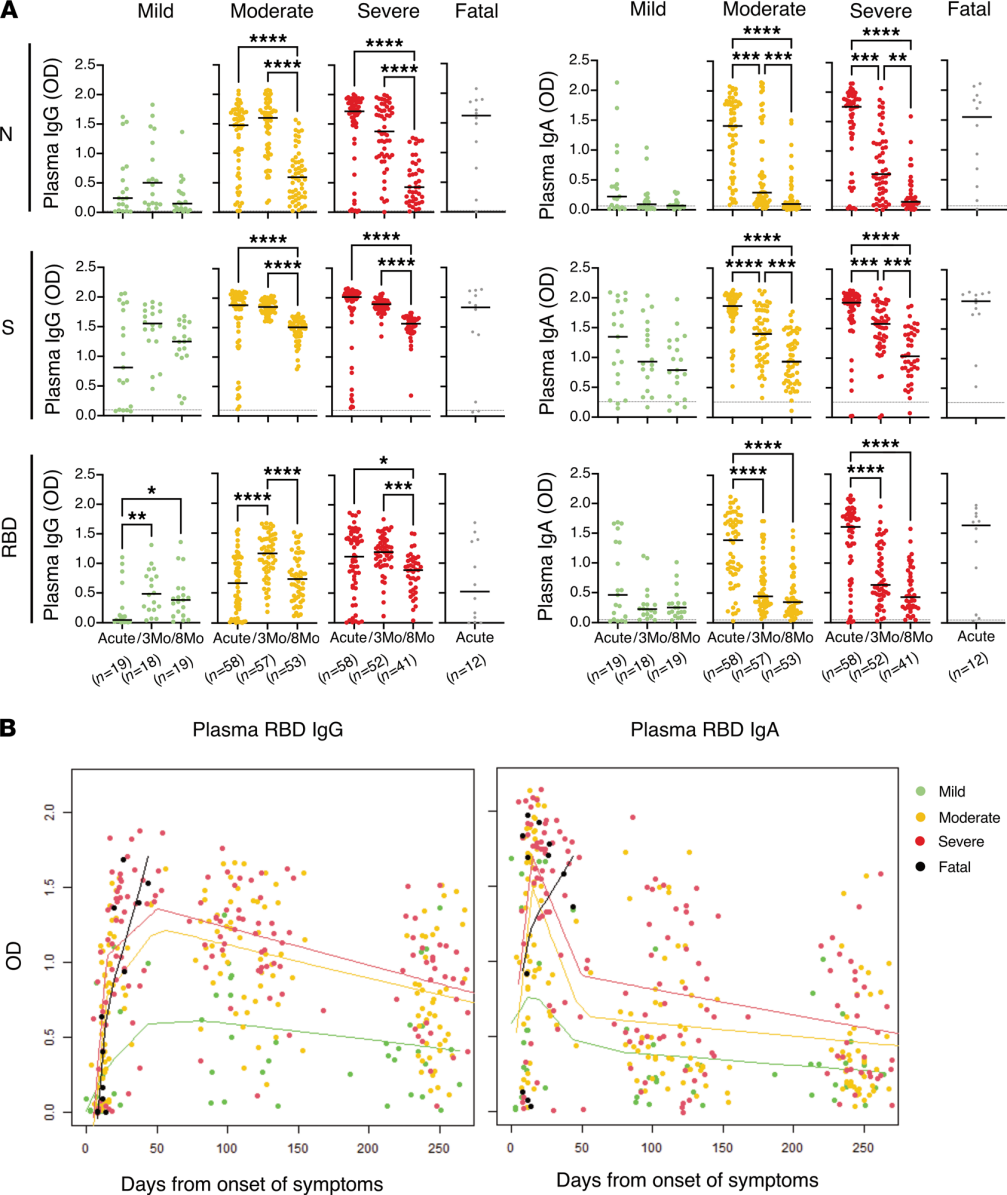
Longitudinal systemic antibody responses across COVID-19 severity from acute disease up to 8 months from symptom onset. (**A**) Individual levels of plasma IgG and IgA (from left to right) in SARS-CoV-2–infected individuals (*n* = 147) with different PDS. Black lines indicate medians and dotted lines indicate the average background level from prepandemic healthy controls. Kruskal-Wallis with Dunn’s multiple comparisons was used to compare the groups and considered statistically significant at *P* < 0.05. *P < 0.05, **P < 0.01, ***P < 0.001, ****P < 0.0001. (**B**) Splines graphs of the plasma RBD IgG and IgA level changes over time (*n* = 19 for mild, *n* = 58 for moderate, *n* = 58 for severe, and *n* = 12 for fatal). All observations are graphed together with kernel-smoothed curves, and data points for each group color-coded as previously with the exception of the “fatal” group, which in this figure is highlighted in black. The bandwidth for the smoothing was set to 40, except for the “fatal” group, for which, due to few and concentrated observations, the bandwidth was set to 10.

We first assessed systemic IgG and IgA responses against N, S, and RBD at the time of study inclusion that ranged between 0 and 54 days from onset of symptoms, with median 16 days for the whole cohort (Table 1). Both IgG and IgA levels against all viral proteins followed the degree of disease severity with increasing levels in patients with mild, moderate, and severe disease, respectively (Figure 2A). In line with previous reports, IgG against N were the most elevated in patients who had severe disease or a fatal outcome (8, 10). The degree of disease severity also associated with the levels of systemic inflammation as indicated by the levels of C-reactive protein (CRP) in blood and by the neutrophil/lymphocyte ratio (NLR) (Figure 2, B and C). Interestingly, the levels of neutrophils also specifically associated with disease severity (Figure 2D) and with all the systemic antibody responses during acute disease (Figure 2D and Supplemental Figure 1; supplemental material available online with this article; https://doi.org/10.1172/jci.insight.151463DS1). The levels of IgG during acute disease, and to a lesser extent IgA, against all tested antigens, exhibited a positive correlation with the days from onset of symptoms (Supplemental Figure 2A). This difference in antibody titers over time might be slightly accentuated by the fact that in our cohort the patients with moderate/severe disease, and even fatal outcome, for whom we initially observed low IgG titers against RBD, had an early study inclusion (on average 13 days from onset of symptoms). In fact, these patients showed significantly higher titers later during the acute phase (on average 19 days) (Supplemental Figure 2, B and C). Nonetheless, patients with mild disease displayed lower levels of plasma IgG against RBD as compared with more severe patients, also when samples were taken after similar duration of symptoms (Supplemental Figure 2D). After 3 months from symptom onset, the IgG levels remained high in the plasma of patients recovering from moderate and severe disease, while the levels had further increased in the individuals who had mild disease (Figure 3A). However, despite this increase over time, the antibody levels in mild patients never reached the levels observed for moderate and severe patients or for those who had a fatal outcome (Figure 3 and Supplemental Figure 3A).

The IgG levels had significantly waned from 3 to 8 months in patients who recovered from moderate and severe disease, but the decline was smaller in patients who experienced mild disease (Figure 3B, Supplemental Figure 3, and Supplemental Table 1). In contrast to IgG, IgA levels from the acute phase, against all antigens, waned substantially in most patients after 3 months (Figure 3, Supplemental Figure 3, and Supplemental Table 1). Antibody titers during acute disease correlated with peak disease severity as well as with disease severity at the time of sampling (Supplemental Figure 4). The correlation between antibody titers and peak disease severity was maintained also when analyzing the antibodies at the 3- and 8-month follow-up visits (Supplemental Figure 4) as also observed in another study (17). Two multivariable linear regression models were also used to estimate the effect of disease severity, days from onset of symptoms, age, sex, and CCI on the different plasma antibody levels during the acute phase. One unadjusted model and a model adjusted for these parameters were used (Supplemental Table 2). The results from these analyses confirmed the relation between antibody titers and severity as well as the relation between antibodies and days from onset of symptoms (Supplemental Table 2).

### Airway IgG and IgA responses and assessment of B cell frequencies in the respiratory tract.

**Figure 4:**
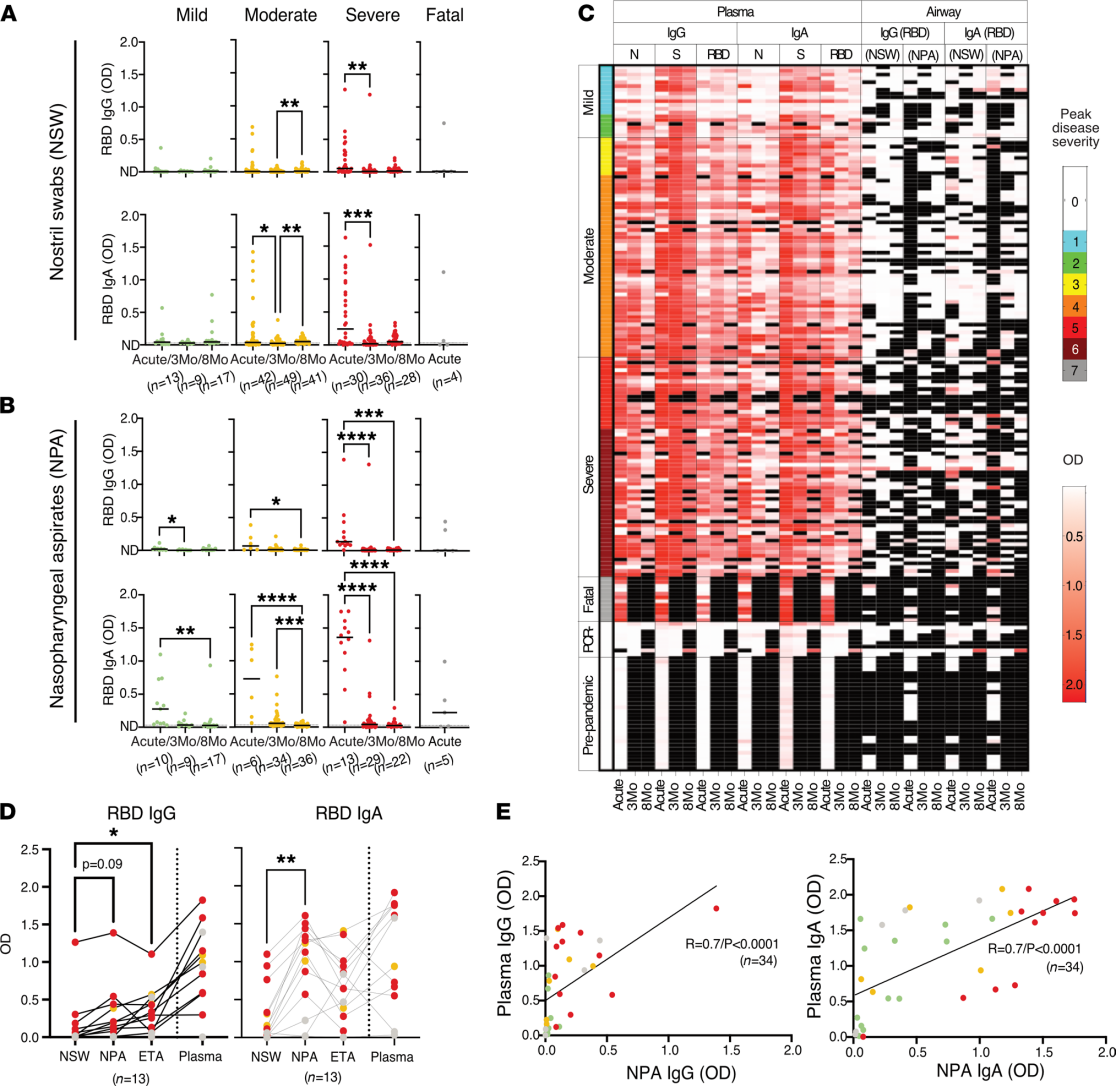
Longitudinal airway antibody responses to RBD across COVID-19 severity from acute disease up to 8 months from symptom onset. Levels of IgG and IgA against RBD in (**A**) NSWs and (**B**) NPAs. The black lines indicate median values. Kruskal-Wallis with Dunn’s multiple comparisons was used to compare the groups and considered statistically significant at *P* < 0.05. *P < 0.05, **P < 0.01, ***P < 0.001, ****P < 0.0001. In **A** the line overlaps with not detected (ND) for IgG levels. (**C**) Heatmap generated grouping patients according to PDS showing acute and convalescent IgG and IgA titers against N, S, and RBD (plasma) and RBCs (NSWs, NPAs, and ETAs) for each patient. The heatmap includes data from patients (*n* = 147) and also data from PPHCs (*n* = 30) and PCR^–^ individuals (*n* = 9) (indicated with PDS 0). Missing data and unavailable samples are shown in black. (**D**) Comparison of the levels of RBD IgG/A in patient-matched NSWs, NPAs, ETAs, and plasma collected at the same time point. The black lines connect data points from the same individuals. Friedman’s test with Dunn’s multiple comparisons test was used to compare the groups and considered statistically significant at *P* < 0.05. *P < 0.05, **P < 0.01. (**E**) Spearman correlation for NPAs (*n* = 34) versus plasma immunoglobulins against the RBD during acute disease.

**Figure 5:**
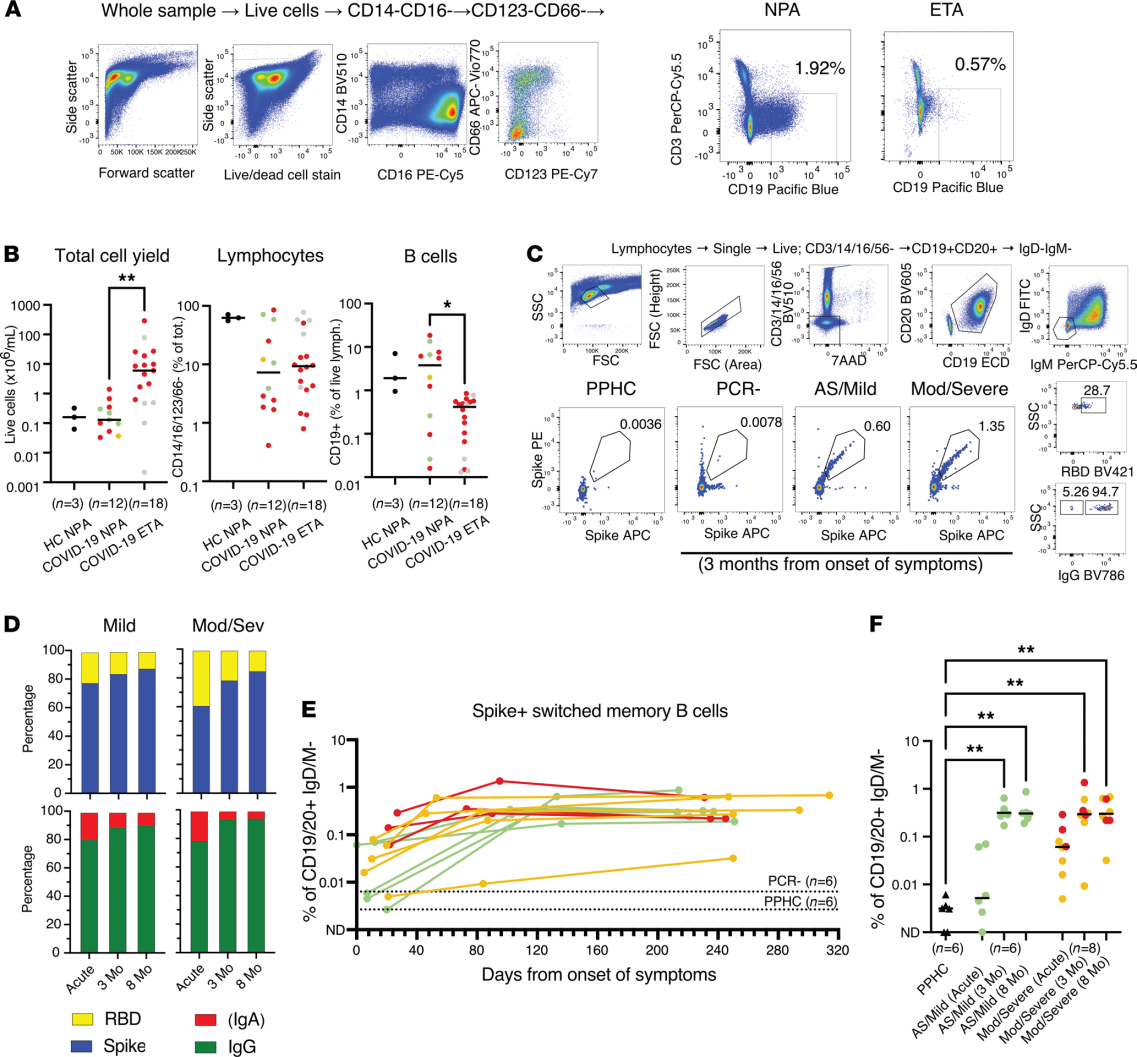
Assessment of frequencies of B cells in the respiratory tract and of circulating S-specific memory B cells. (**A**) Representative example with gating strategy for the identification of lymphocytes (identified as negative for CD14/16/123/66) and of total B cells (CD3^–^CD19^+^) in respiratory NPA and ETA samples. (**B**) Lymphocytes and total B cells in NPAs and ETAs in a subset of patients alongside NPAs from healthy controls. Kruskal-Wallis with Dunn’s multiple comparisons test was used and considered statistically significant at *P* < 0.05. *P < 0.05, **P < 0.01. (**C**) Representative examples with gating strategy of SARS-CoV-2 S-specific memory B cells from 1 PPHC and 3-month follow-up samples from 1 SARS-CoV-2 PCR^–^ individual and 1 mild and 1 moderate/severe COVID-19 patient. Further characterization of S-positive memory B cells on RBD binding and B cell isotype (IgG^+^ or IgA^+^ assumed to correspond to IgD^–^IgM^–^IgG^–^ B cells). (**D**) Bar charts show the cumulative proportion (frequency) of S- (blue) and RBD- (yellow) specific memory B cells as well as the proportion of IgG (green) versus IgA (red) isotypes among the S-specific memory B cells in longitudinal samples from mild (*n* = 6) and moderate/severe (*n* = 8) COVID-19 patients. (**E**) Frequencies of S-specific memory B cells in matched acute and 3-month follow-up PBMCs in relation to days in the subset of individuals analyzed (*n* = 14) color-coded according to PDS. Dotted lines indicate the average background staining from PCR^–^ and PPHC. (**F**) Levels of circulating S^+^ switched memory B cells during acute disease and convalescence in the subset of patients analyzed, as well as PPHCs, color-coded according to PDS. Black triangles symbolize the PPHCs. Differences were assessed using Kruskal-Wallis with Dunn’s multiple comparisons test and considered statistically significant at *P* < 0.05. **P < 0.01.

We next measured the levels of IgG and IgA in the upper and lower airways and compared them with levels in plasma at matched time points. Due to limited respiratory sample volumes, we focused our analyses on IgG and IgA responses against the RBD since these responses are most critical for virus neutralization. We found that RBD-specific antibodies could be detected in NSWs (Figure 4A) and NPAs (Figure 4B) during the acute phase across all disease severities (Figure 4, A–C). In agreement with our observations in plasma, antibody levels in the upper respiratory tract were higher in patients with moderate or severe disease as compared with individuals with mild disease. Both IgG and IgA levels had declined significantly already after 3 months, with IgG declining to almost undetectable levels (Figure 4, A–C). RBD antibody levels during acute infection were on average higher in NPAs compared with NSWs for both IgG and IgA across disease severity (Figure 4, A–C), suggesting that antibody titers may increase not only with disease severity but also with sampling at different depths of the upper airway. To address this, we compared the antibody content between donor-matched NSWs (peripheral nostril), NPAs (upper airway), and ETAs (trachea) collected at the same time point during acute disease from intubated patients from whom we had peripheral and upper and lower airway samples. Interestingly, we still found significantly higher levels of IgA against the RBD in NPAs as compared with NSWs and ETAs (Figure 4D). Furthermore, nasopharyngeal antibody levels (both IgG and IgA) showed a strong correlation with plasma antibody responses (Figure 4E). We also assessed the presence of B cells in the respiratory tract of patients with COVID-19 by analyzing the lymphocytes that could be retrieved from NPAs and ETAs as compared with NPAs from 3 healthy controls (HCs). Despite generally obtaining a significantly lower cell yield from NPAs as compared with ETAs, lymphocyte frequencies did not differ in NPAs and ETAs from patients with COVID-19, but both were lower as compared with NPAs from HCs. Instead, the proportion of B cells in NPAs was higher as compared with ETAs in patients with COVID-19 and similar to NPAs from HCs (Figure 5, A and B).

### Expansion of SARS-CoV-2–specific memory B cells.

As mentioned above, the virus-specific B cell memory pool will be essential to remount a rapid antibody response in the case of reexposure. To assess the establishment of antigen-specific memory B cells, donor-matched PBMCs from acute disease and convalescence were analyzed side by side using fluorescently labeled S and RBD probes (18–20). Patients with moderate/severe disease showed the presence of Ig-switched memory B cells specific to S in the acute phase, and the memory B cell pool had further expanded after 3 months (ranging from 0.009% to 1.35%; mean 0.42% during convalescence) (Figure 5, C–F). Individuals with mild disease showed lower frequencies of S-specific memory B cells during acute disease than the patients with moderate/severe disease. In fact, the frequencies of S-specific memory B cells in the patients with mild disease during the acute phase were not different from those observed in the PCR^–^ individuals or in the PPHCs (Figure 5, C and E). However, the frequencies of S-specific memory B cells had substantially increased in the patients with mild disease after 3 months (ranging from 0.17% to 0.64%; mean 0.35% during convalescence) and were comparable to frequencies among severely ill patients. In addition, the levels were well maintained between 3 and 8 months in all groups (Figure 5, E and F). Further phenotyping of the S-specific memory B cells indicated that the majority of these cells may be specific for epitopes on S outside of the RBD (Figure 5D). S-specific memory B cells in the circulation were predominantly IgG^+^, rather than IgA^+^ (Figure 5D).

### The effect of vaccination on systemic and airway antibody levels.

**Figure 6:**
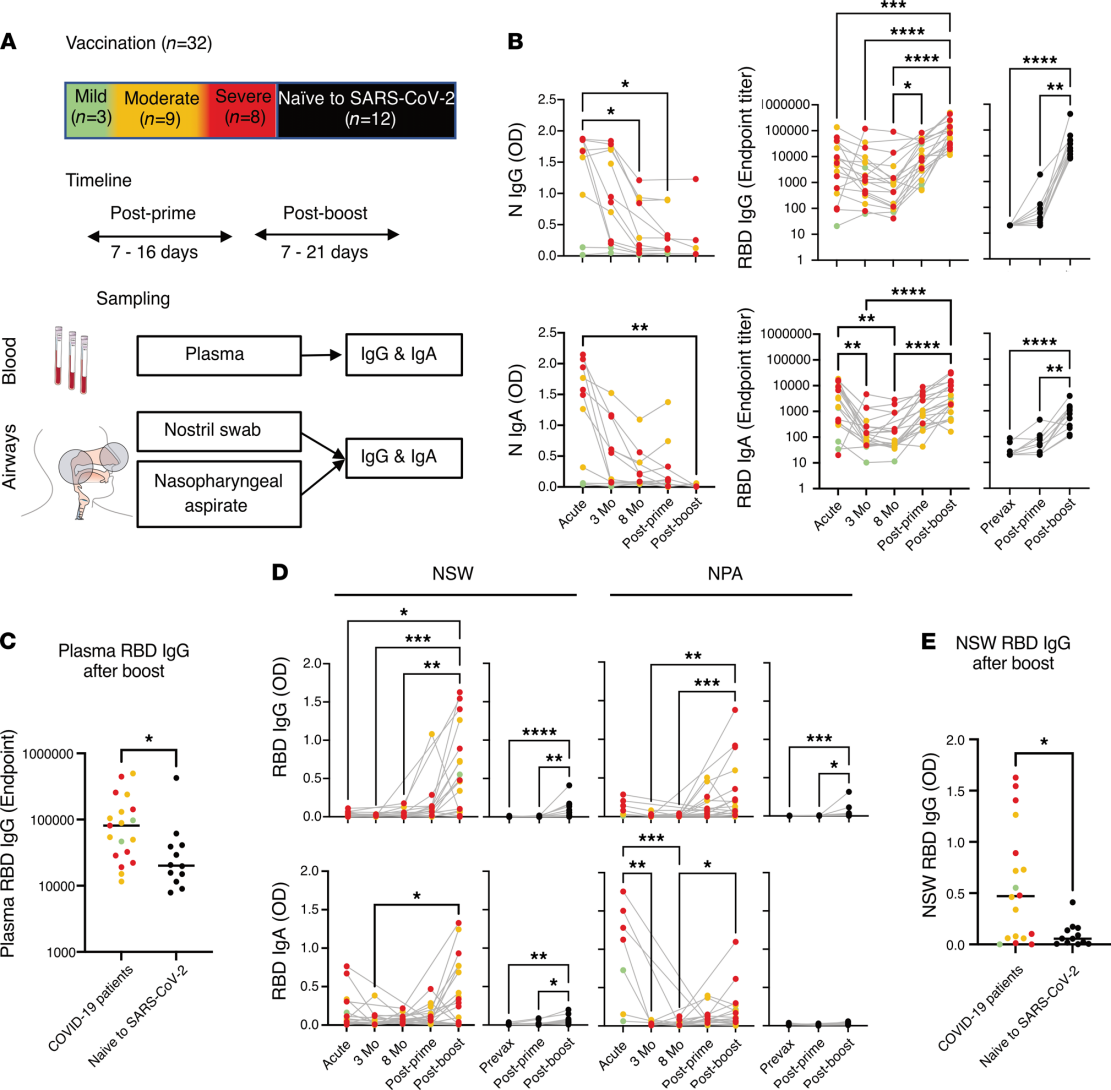
Vaccination and systemic and airway antibody level rebound. (**A**) Overview of vaccinated patients (*n* = 20) with respect to PDS during COVID-19 and sampling timeline after prime and boost as compared with vaccination in individuals naive to SARS-CoV-2 (*n* = 12). The anatomical compartments analyzed and the measurements performed are also shown. (**B**) Compiled patient-matched longitudinal data from acute, 3-month, and 8-month follow-ups are shown together with data from after prime and after boost for the levels of plasma IgG and IgA against N and RBD. (**C**) Direct comparison between plasma RBD IgG after boost in patients with COVID-19 and individuals naive to SARS-CoV-2. (**D**) Compiled data as above for RBD IgG and IgA in NSWs and NPAs. (**E**) Direct comparison between NSW RBD IgG after boost in patients with COVID-19 and individuals naive to SARS-CoV-2. The gray lines connect data points from the same individuals. Data are color-coded according to PDS during COVID-19, with data from individuals naive to SARS-CoV-2 shown in black as a comparison. Differences were assessed using Kruskal-Wallis with Dunn’s multiple comparisons test and considered statistically significant at *P* < 0.05. *P < 0.05, **P < 0.01, ***P < 0.001, ****P < 0.0001.

We finally evaluated the influence of SARS-CoV-2 vaccination on the systemic and airway antibody responses (Figure 6A). A subset of 20 individuals, 3 who recovered from mild, 9 from moderate, and 8 from severe COVID-19 a year earlier, were sampled after receiving their scheduled vaccination (range 270–407 days; median 339 days from symptom onset) (Table 2). Donor-matched plasma, NSWs, and NPAs were collected at different time points after prime (7–16 days) from 18 patients and after boost (7–28 days) from 19 patients alongside with samples from 12 individuals naive to SARS-CoV-2 (7–10 days after prime and boost vaccinations) to be included as a reference control. All samples were analyzed for the presence of IgG and IgA against RBD. Antibodies against N were also measured in patient plasma as a negative control as the vaccines used were based on the S protein. After vaccination, all individuals demonstrated a significant increase of both plasma IgG and IgA against RBD (Figure 6B) but, as expected, not against N (Figure 6B). While the IgG levels to RBD after boost vaccination exceeded the levels detected during the acute phase, the IgA levels were equally high (Figure 6B). On the contrary, individuals naive to SARS-CoV-2 only had a moderate increase of IgA as compared with IgG after boost (Figure 6B). IgG levels after boost were significantly lower in individuals naive to SARS-CoV-2 as compared with those from patients with COVID-19 after boost (Figure 6C and Supplemental Figure 5A). The airway IgG levels against RBD also showed a noticeable increase after the boost vaccination in particular. In fact, the IgG levels in the airway samples, both NSWs and NPAs, were in many individuals significantly higher after boost vaccination than they were in the acute stage of the disease (Figure 6D). In contrast, this was not noted for IgA levels against RBD (Figure 6D). On the other hand, individuals naive to SARS-CoV-2 had a modest but significant increase of IgG in NSWs and NPAs and of IgA in NSWs after boost (Figure 6D). Despite IgG levels in NSWs having the highest increase after boost in individuals naive to SARS-CoV-2, levels were generally significantly lower as compared with those from patients with COVID-19 (Figure 6E and Supplemental Figure 5A).

## Discussion

By now, it is well documented that higher systemic antibody levels are generated in severe as compared with mild COVID-19 (7–11, 21–23). In contrast, the presence and durability of antibodies against SARS-CoV-2 in the airways are much less understood. Whether and how respiratory antibody levels are influenced by SARS-CoV-2 vaccination in humans are unknown. In this study, we performed longitudinal analyses of systemic and upper and lower airway antibody responses in a clinically well-characterized and relatively large cohort of individuals with SARS-CoV-2 infection representing the full spectrum of COVID-19 severity ranging from asymptomatic infection to fatal disease. Matched analyses in blood and in the airways enabled us not only to address the magnitude and durability of systemic antibodies to SARS-CoV-2 but also to gain insights into the prospects of protective capacity locally in the mucosa at virus reentry. This is one key aspect still largely unknown yet critical for our understanding of immunity to and protection from SARS-CoV-2. Furthermore, we studied how the systemic versus airway antibody levels were affected by vaccination. Collectively, these data will contribute to a better understanding of long-term protective effects and whether vaccination is important to boost the capacity of virus neutralization in the airways and thus reduce reinfection and virus spread.

Airway mucus along the respiratory tract is thought to serve as a barrier that can trap respiratory viruses via virus glycoprotein-mucin interactions (24). However, it has been shown that local immobilization of respiratory viruses such as influenza viruses in the airways mostly occurs by binding with virus-specific antibodies present in the mucus (25). As the respiratory tract is the initial site of viral infection and replication, the levels of IgG and IgA against the RBD in the upper and lower airways are likely critical for SARS-CoV-2 neutralization and could therefore help predict the ability of individuals to neutralize the virus locally in case of reexposure. Low but detectable levels of antibodies against SARS-CoV-2 have previously been reported in saliva during convalescence (26). However, measurements of antibodies in saliva may primarily represent plasma exudate from the gingiva (27) while respiratory secretions better reflect the mucosal responses. Sampling the respiratory mucosa is indeed more likely to be sensitive to sampling methods compared with blood draws. Ideally it is therefore important to sample multiple compartments to more comprehensively understand the immunity to SARS-CoV-2. In our study we found that IgG and IgA against the RBD can be readily detected in the upper and lower airways during acute disease and that such levels correlated with the systemic response at the same time point and also followed disease severity. However, for all the patients across disease severities, airway antibodies waned to low levels much faster than those in plasma during convalescence. Whether these low antibody levels observed at respiratory sites will be sufficient for preventing virus reentry or for protection is not known. The correlation between systemic and airway antibody levels during acute disease raises questions on whether the low levels of antibodies in the airways during convalescence are due to decreased antibody generation locally at mucosal sites or are rather caused by decreased dissemination from the periphery once systemic antibody levels start to wane. Antibodies in the upper respiratory tract have been shown to be dominated by secretory IgA, which are mostly produced by plasma cells in the lamina propria of mucosa-associated lymphoid tissue (MALT) (28, 29). We detected high levels of IgA in the upper airways early during acute COVID-19 that rapidly declined during convalescence, following the pattern observed for systemic IgA levels here and in other reports (30–32). This suggests that at least some IgA disseminated into the airways from the circulation. In contrast, the dynamics of IgG were different in the respiratory samples compared with plasma, with airway IgG following the same kinetics as IgA, while systemic IgG levels were well maintained up to 8 months.

When we assessed the presence of lymphocytes in the different airway compartments during acute disease, we observed a higher proportion of B cells along with high antibody levels, especially IgA, in the nasopharynx, as compared with the nostril or the endotracheal space. It has previously been shown that the majority of antibody-secreting cells generated after intranasal immunization with live-attenuated vaccines in rodents may reside in the respiratory tract rather than in the spleen and bone marrow (33) and that these cells secrete IgA early after a later challenge with the vaccination pathogen (34–36). Therefore, it is possible that B cells generated during SARS-CoV-2 infection also reside locally in the airways and contribute to antibody levels in the nasopharynx. While the antibody content in NPAs and ETAs could be influenced by differences in sampling methods and sample volumes, these data suggest that antibody abundance and possibly virus neutralization via IgA differ along the respiratory tract and may be more pronounced in the nasopharynx compared with the lower airways. Altogether, our observations demonstrate that moderate and severe COVID-19 result in high levels of circulating antibodies and despite IgG levels being well maintained, antibody levels in the airways decline to almost undetectable levels after the acute phase.

Once antibody titers have waned below protective levels, the response to a secondary infection will mostly rely on the presence of resting antigen-specific memory B cells that can rapidly activate upon antigen reexposure (13). Therefore, similar to other studies (18–20), we investigated the induction and maintenance of S-specific memory B cells. Importantly, because of the comprehensive range of disease severity represented in our cohort, we were able to compare the opposite ends of the COVID-19 disease spectrum by focusing on individuals with mild disease as compared with patients with moderate/severe disease, who had the highest circulating IgG and IgA levels. Strikingly, despite the fact that these patients were at opposite ends of the disease severity spectrum, they had comparable levels of S-specific memory B cells during convalescence. These appeared to be specific for epitopes on S outside the RBD and were predominantly IgG^+^, rather than IgA^+^, which may affect the proportions of different isotypes subsequently produced in the event of antigen reexposure.

Immunization at mucosal sites such as, for example, intranasal administration of live-attenuated influenza vaccines generally elicits mucosal immune responses (37). However, several studies, primarily performed with DNA and virus-like particle vaccines, have shown that intradermal, subcutaneous, and intramuscular immunization also can result in local mucosal responses that protect from mucosal challenge (38). It has been speculated that this could be due to free antigen or B cells migrating from the vaccine draining lymph nodes to the MALT (38–40). A 2-dose regimen of Moderna’s mRNA-1273 vaccine administered intramuscularly and followed by intranasal and intratracheal challenge with SARS-CoV-2 in rhesus macaques has indeed resulted in local virus neutralization in the airways (41). Antibodies in bronchoalveolar lavage and NSWs were elicited in a vaccine dose-dependent manner assessed after the boost vaccination (42).

Whether the systemic and/or mucosal immunity generated during natural infection is boosted by vaccination and results in a similar or enhanced magnitude of responses would be important knowledge to acquire for planning the best vaccination strategies for SARS-CoV-2 as well as for other respiratory viruses. Our results from individuals recovering from COVID-19 and subsequently receiving vaccination indicated a marked increase of both IgG and IgA levels systemically but also strikingly in the airways, which in the majority of cases exceeded the levels observed during acute disease. In contrast, vaccination of individuals naive to SARS-CoV-2 only resulted in a modest increase of airway antibodies, mainly IgG, after boost vaccination. Notably, the antibody increase observed between prime and boost vaccination in the patients was more prominent in the airways than systemically. Recent studies on systemic antibody responses after SARS-CoV-2 vaccination in individuals who recovered from COVID-19 have shown a significant increase in antibody levels after 1 vaccine dose with no or only a small increase after the second dose (43–47). This suggests that 1 vaccine dose may be sufficient to protect these individuals from disease in case of reinfection, which is important for vaccine dose management at the population level. However, our data indicate that only assessing the systemic antibody levels after vaccination is to some extent misleading as respiratory antibody levels, and likely virus neutralization, may be substantially better with a prime-boost vaccination strategy rather than with 1 single dose. Two earlier studies have been able to demonstrate neutralizing activity of antibodies in the upper respiratory tract after vaccination in individuals who earlier had COVID-19 (48, 49).

The higher levels of airway antibodies that we observed after 2 vaccine doses may be explained by even a small increase in circulating antibodies after the boost causing a substantial extravasation from the bloodstream into mucosal sites (Supplemental Figure 5B). On the other hand, the fact that naive individuals had less pronounced airway antibodies after vaccination despite eliciting relatively high plasma antibody levels suggests that airway antibody responses are better primed with natural infection and that vaccination after COVID-19 stimulates anamnestic local mucosal responses. It remains to be investigated how airway antibodies induced after intranasal vaccination would compare to natural infection and whether an intramuscular vaccine boost would affect these responses (50).

In summary, here we show that COVID-19 disease severity not only determines the magnitude of systemic but also airway antibody levels with efficient generation of virus-specific memory B cells against SARS-CoV-2 also occurring upon mild disease. While plasma IgG levels were generally detectable after acute disease in all groups, there was a significant decline in airway antibodies during convalescence. This suggests that antibodies in the airways may not be maintained at levels that prevent local virus entry upon reexposure. However, our data indicate that the majority of infected individuals have the ability to generate anamnestic responses via the memory B cell pool and that vaccination against SARS-CoV-2 resulted in a substantial rebound of both systemic and airway antibodies in patients who recovered from COVID-19. These data indicate a positive effect of vaccination for increased virus neutralization in the airways and prospects of reduced virus spread, which further supports following the full vaccination schedule also in this population.

## Methods

### Study design, patient enrollment, and sample collection.

One hundred forty-seven PCR-confirmed SARS-CoV-2–infected patients were enrolled at the Karolinska University Hospital and Haga Outpatient Clinic (Haga Närakut), Stockholm, Sweden, during March–May 2020 (acute phase) in a time that ranged from 0 to 54 days from onset of symptoms as self-reported by individual patients and during April–September 2020 (3 months) in a time that ranged from 46 to 168 days and during November 2020 to February 2021 (8 months) continuing from the previous counts. Patients were enrolled at various settings, ranging from primary to intensive care. In order to recruit asymptomatic and mild cases, household contacts of patients with COVID-19 were enrolled and screened with PCR to identify SARS-CoV-2–positive individuals. A small subset of these individuals who experienced influenza-like symptoms and were possibly exposed to SARS-CoV-2 but had a negative diagnostic PCR (*n* = 9, of whom 3 were household contacts of confirmed patients with 1 experiencing fever, and 6 were included based on suspected infection with 4 experiencing fever) were sampled in the same way and included as controls alongside 30 PPHCs from 2016 to 2018. Twelve individuals naive to SARS-CoV-2 were also recruited at the Umeå University, Umeå, Sweden, as a control group for vaccination (Table 2). These individuals were identified as naive to SARS-CoV-2 based on lack of COVID-19 symptoms and of positive diagnostic PCR test throughout the pandemic in combination with absence of plasma antibodies against SARS-CoV-2 prior to vaccination.

Respiratory failure was categorized daily according to the respiratory domain of the SOFA (14). The modified SOFA score (mSOFA) was calculated when arterial PaO_2_ was not available. In this case peripheral transcutaneous hemoglobin saturation (SpO_2_) was used instead (15). Estimation of the FiO_2_ based on O_2_ flow was calculated as per the Swedish Intensive Care register definition (51). Patients were categorized based on the peak respiratory SOFA or mSOFA value with the 4-point respiratory SOFA score being extended with additional levels to distinguish between admitted and nonadmitted mild cases (both respiratory SOFA score 0) and to include fatal outcome. Ten patients with fatal outcome had peak disease severity score 6 prior to death, and 2 patients had scores of 4 and 5. For convenience, the resulting 7-point composite peak disease severity was condensed into a broader classification consisting of mild, 1–2; moderate, 3–4; severe, 5–6; and fatal, 7. Demographics and additional data were collected from medical records, including clinical history and risk factors such as BMI and comorbidities. Total burden of comorbidities was assessed using the CCI (52) (Table 1). Additional clinical information on this patient cohort including the modulation of disease from time to study inclusion to peak severity can be found in Falck-Jones et al. (16).

Blood was collected in EDTA-containing tubes from all patients except those admitted to the intensive care unit (ICU), for whom blood was pooled from heparin-coated blood gas syringes discarded in the last 12 hours. For some ICU patients, additional venous blood was also collected in EDTA tubes. NSWs and NPAs were collected from the majority of the patients whereas ETAs were only collected from patients with mechanical ventilation intubated in the ICU. Admitted patients were sampled during acute disease at up to 4 time points, and ICU patient material was collected up to 10 time points. For this study, unless otherwise stated, the measurements referring to acute disease were performed with samples collected at the time of study inclusion and when patients returned for their follow-up visits at 3 and 8 months from symptom onset. At follow-up sampling, all study individuals had been discharged (if hospitalized) from the infectious diseases ward, but some individuals (<10) who recovered from severe COVID-19 were still in a hospital aftercare ward at the first follow-up sampling. All study participants were confirmed SARS-CoV-2 negative by PCR at the time of follow-up sampling, with the exception of 5 individuals who were PCR^+^ but with high Ct values (>34).

### Enzyme-linked immunosorbent assay.

The presence of IgG or IgA binding against the SARS-CoV-2 N and S trimer or the RBD monomer (5, 6) in plasma and airway samples was assessed by enzyme-linked immunosorbent assay (ELISA). Recombinant proteins were received through the global health-vaccine accelerator platforms funded by the Bill & Melinda Gates Foundation, Seattle, Washington, USA. Briefly, 96-half-well plates were coated with 50 ng/well of the respective protein. Plates were incubated with a selected duplicate dilution that did not provide background noise against ovalbumin used as a negative control (data not shown) (i.e., 1:20 for plasma samples, 1:2 for NSWs and NPAs, and 1:5 for ETAs in 5% milk/PBS buffer). Duplicate 7-point serial dilutions were initially performed for measuring plasma IgG against RBD during acute disease and after vaccination. The half maximal effective concentration (EC_50_) or the endpoint titer (dilution at the set OD value of 0.1) were calculated using GraphPad Prism 9. The relation between EC_50_ and endpoint titer for these samples is shown in Supplemental Figure 5C. However, since for several samples with low antibody concentration (mostly from the asymptomatic/mild category), the EC_50_ was below the highest dilution used (of 1:20) and therefore below the limit of detection (Supplemental Figure 6A), the maximal OD at 1:20 dilution was used for most of the analyses. The relation between maximal OD and EC_50_ was verified in a subset of patients with high IgG and IgA against S (Supplemental Figure 6B). To be able to compare pre- and postvaccination antibody levels that would, in some instances, fall below and above the lower and upper limits of detection, the endpoint titer was used instead. Detection was performed with mouse and goat anti–human IgG or IgA HRP-conjugated secondary antibodies (clone G18-145 from BD Biosciences and polyclonal from Thermo Fisher, respectively) followed by incubation with TMB substrate (BioLegend), which was stopped with a 1 M solution of sulfuric acid. Blocking with 5% milk/PBS buffer and washing with 0.1% Tween 20/PBS buffer were performed between each step. Absorbance was read at 450 nm and background correction at 550 nm using an ELISA reader. Data were reported as maximal absorbance, i.e., OD, as stated above, and plotted using GraphPad Prism 9. All the antibody measurements in plasma and respiratory samples from patients with SARS-CoV-2 were run alongside samples from 2 different control groups as described above. Interestingly, low but readily detectable IgA reactivity against S was detected in the PPHCs and in the PCR^–^ individuals (Supplemental Figure 6C). Having verified the specificity and sensitivity of our ELISA for IgA detection with limiting sample dilutions (Supplemental Figure 6, D and E), we hypothesize that this might be due to cross-reactivity on the shared portions of the S protein between SARS-CoV-2 and other common cold coronaviruses. Reports have shown that cross-reactivity between coronaviruses exists (53, 54).

### Flow cytometry.

Staining of cells from airway samples was performed. Briefly, samples were centrifuged at 400*g* for 5 minutes at room temperature and cells were washed with sterile PBS. Mucus was removed using a 70 μm cell strainer, and cells were subsequently stained with the appropriate combination of fluorescently labeled monoclonal antibodies as illustrated in Figure 5A and in Table 3. Staining of PBMCs was performed on previously cryopreserved samples. The appropriate combination of fluorescently labeled monoclonal antibodies binding to different cell surface markers and with fluorescently labeled S and RBD proteins used as probes for antigen-specific B cells is illustrated in Figure 5C and in Table 4. Probes were prepared from biotinylated proteins using a 4:1 molar ratio (protein/fluorochrome-labeled streptavidin) considering the molecular weight of protein monomers and of the streptavidin only. The probes were prepared using streptavidin conjugated to PE and APC for S and with BV421 for the RBD. The gating strategy for the identification of antigen-specific memory B cells is shown in Figure 5C. Briefly, after identification of lymphocytes in single suspension, live B cells (i.e., cells not expressing CD3/CD14/CD16/CD56) were gated. From this gate, B cells were further isolated by expression of CD19 and CD20, and then switched memory B cells were identified as IgD^–^IgM^–^. From these, S-specific switched memory B cells were identified by binding to both S protein probes. Further characterization was then carried out by analyzing IgG expression (IgA^+^ switched memory B cells are assumed to mirror IgD^–^IgM^–^IgG^–^ B cells) and fluorescently labeled RBD. Stained cells from airway samples were acquired using a BD LSRFortessa while stained PBMCs were acquired using a BD FACSAria Fusion both interfaced with the BD FACSDiva software. Results were analyzed using BD FlowJo version 10.

### Statistics.

Spearman correlation was used to assess the interdependence of 2 different noncategorical parameters across individuals whereas Wilcoxon matched pairs signed-rank or Mann-Whitney *U* tests, as appropriate, were used to assess differences or similarities for 1 single parameter between 2 different groups. Kruskal-Wallis with Dunn’s multiple comparisons test was used when comparing between multiple groups. All of the above statistical analyses were performed using GraphPad Prism 9.

The effect of disease severity on the acute response was estimated using linear regression. We estimated both unadjusted models, as well as models adjusted for age, sex, days from onset of symptoms, and CCI. The longitudinal models using splines were estimated using multivariate multiple regression. The splines used were linear, with knots placed on days 15 and 50. The location of the knots was chosen based on visual inspection of the data, aided by kernel smoothing. The effect on standard deviations from repeated measures was not adjusted for, as the primary focus of the longitudinal analysis was description rather than statistical testing. Analysis was done in R, version 4.1.0.

When not stated otherwise, *P* values less than 0.05 were considered statistically significant.

### Study approval.

The study was approved by the Swedish Ethical Review Authority and performed according to the Declaration of Helsinki. Written informed consent was obtained from all patients and controls. For sedated patients, the denoted primary contact was contacted and asked about the presumed will of the patient and to give initial oral and subsequently written consent. When applicable, retrospective written consent was obtained from patients with nonfatal outcomes.

## Author contributions

AC, K Loré, and ASS designed experiments. ASS, MB, NJ, JA, JS, AF, and MNF designed the clinical concept. AC, MY, SFJ, SV, BÖ, EÅ, LA, RFJ, MÖ, FG, JS, ME, JN, and CA acquired and processed samples. AC, MY, SFJ, SV, LA, and PCG generated data. DDP, MPM, LC, and NPK provided custom reagents. AC, K Loré, and ASS analyzed and interpreted data. All authors critically revised the manuscript. AC, AW, K Lenart, and SO performed statistical analysis. K Loré and ASS contributed equally to the study.

## Supplementary Material

Supplemental data

## Figures and Tables

**Table 4:**
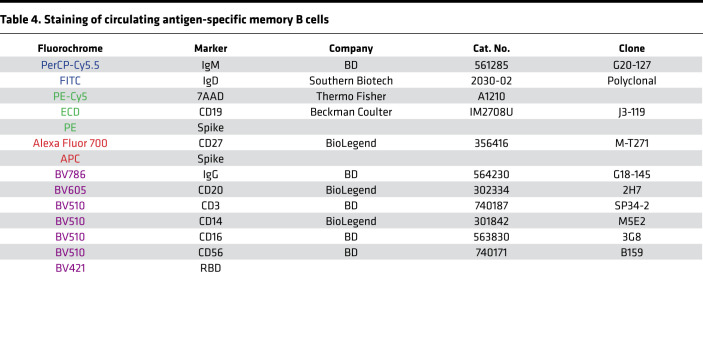
Staining of circulating antigen-specific memory B cells

**Table 3:**
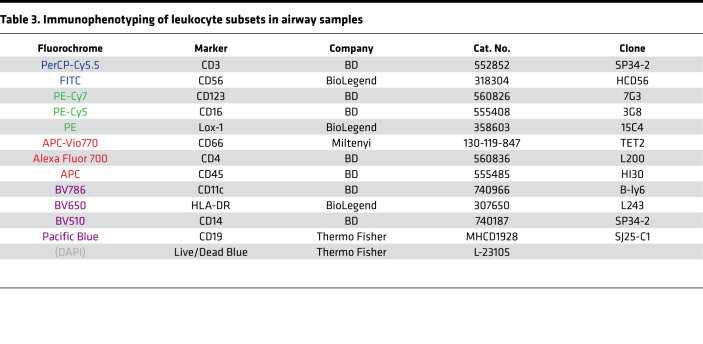
Immunophenotyping of leukocyte subsets in airway samples

**Table 1:**
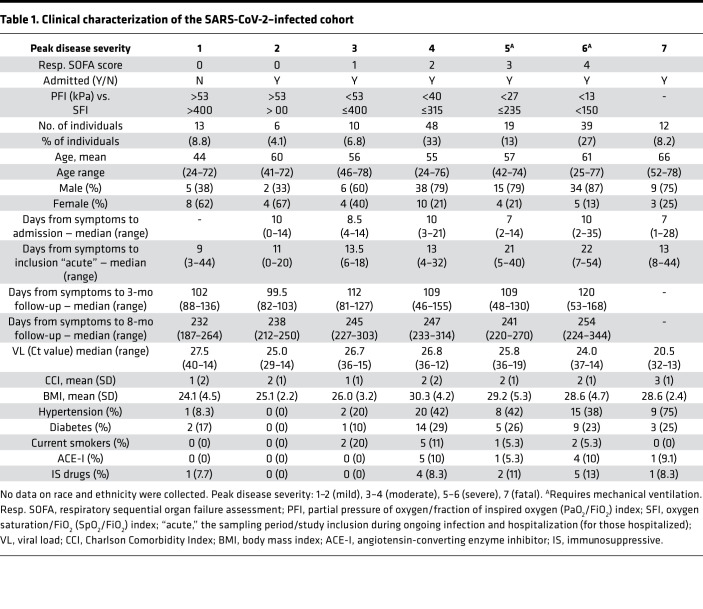
Clinical characterization of the SARS-CoV-2–infected cohort

**Table 2:**
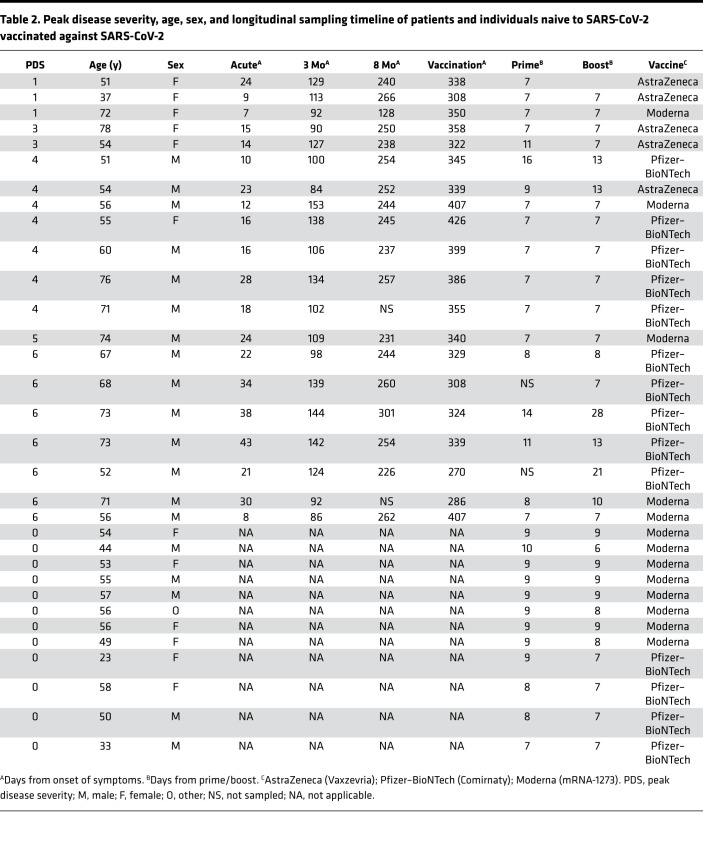
Peak disease severity, age, sex, and longitudinal sampling timeline of patients and individuals naive to SARS-CoV-2 vaccinated against SARS-CoV-2

## Version 1 10/19/2021

In-Press Preview

## Version 2 11/22/2021

Electronic publication
